# Perioperative Dyselectrolytemia in Patients Undergoing Transurethral Resection of the Prostate Using 0.9% Normal Saline Irrigation

**DOI:** 10.7759/cureus.59976

**Published:** 2024-05-09

**Authors:** Surya P Singh, Hiranmay Barman, Reena, Amrita Rath, Rambadan Singh

**Affiliations:** 1 Anaesthesiology, Apex Hospital, Varanasi, IND; 2 Anaesthesiology, Anandaloke Multispeciality Hospital, Siliguri, IND; 3 Anesthesia, Critical Care and Pain Management, Institute of Medical Sciences, Banaras Hindu University, Varanasi, IND

**Keywords:** transurethral resection of the prostate, turp, normal saline, irrigation fluids, hematocrit, dyselectrolytemia

## Abstract

Background: The choice of irrigation fluid used in transurethral resection of the prostate (TURP) has a significant impact on serum electrolyte levels. Among the many available options, 0.9% normal saline (NS) is considered to be more physiological.

Material and methods: This observational study was conducted on 60 adult males aged 50-70 years, classified as American Society of Anesthesiologists grade 1 and 2, undergoing TURP with 0.9% NS irrigation under spinal anesthesia achieved with a mixture of 0.5% heavy bupivacaine. The patients' hematocrit and serum electrolyte levels were obtained after six hours and compared with preoperative values.

Results: Hematocrit reduced from 40.32 ± 6.27 to 31.07 ± 5.40 (p < 0.001). Both serum sodium and potassium decreased from 136.77 ± 3.27 to 128.31 ± 5.91 and from 4.02 ± 0.26 to 3.81 ± 0.36, respectively (p < 0.001). However, serum chloride showed only a minimal increase from 101.58 ± 2.88 to 102.25 ± 1.66 (p < 0.12).

Conclusion: Although the changes in serum sodium and potassium were statistically significant, they did not have any physiological consequences in our study. However, this emphasizes the importance of vigilant electrolyte monitoring to identify and mitigate the risk of electrolyte disturbances during TURP surgeries.

## Introduction

Transurethral resection of the prostate (TURP) remains a cornerstone in the surgical management of benign prostatic hyperplasia (BPH), particularly in elderly patients. Despite advancements in understanding and various minimally invasive alternatives, TURP continues to hold its position as the gold standard for alleviating obstruction secondary to BPH [1]. However, like any surgical procedure, TURP is not devoid of complications, particularly in older patients with multiple comorbidities. Early postoperative complications such as bleeding necessitating transfusion, acute kidney injury, and transurethral resection syndrome significantly impact morbidity and, in severe cases, may even lead to mortality [2,3].

Over the years, advancements in surgical techniques, perioperative management, and anesthetic practices have contributed to a substantial decrease in the incidence of early complications associated with TURP [4,5]. Nonetheless, one of the most concerning complications remains the alteration of electrolyte levels in the blood, primarily due to the risk of developing TURP syndrome. This syndrome ensues from the absorption of irrigating fluid through prostatic veins exposed during the resection process. The irrigating fluid, when absorbed via open venous channels, can lead to hypervolemic hyponatremia, culminating in a spectrum of symptoms, including mental confusion, bradycardia, hypotension/hypertension, nausea, vomiting, and visual disturbances [6,7]. These manifestations primarily stem from the resultant brain edema induced by the hypervolemic hyponatremic state. Additionally, hyperkalemia may occur post-TURP, primarily attributed to cell lysis and the subsequent release of intracellular potassium.

Despite advancements in understanding and technological advances, such as the development of continuous-flow resectoscopes and the utilization of non-hemolytic solutions, the incidence of TURP syndrome still persists, albeit at a reduced rate [8,9]. Identified risk factors associated with TURP include the volume and type of irrigation fluid used, resection time, and the weight of tissue resected [10].

Given the paramount importance of patient safety, our study aims to evaluate perioperative dyselectrolytemia in patients undergoing TURP using 0.9% normal saline as irrigation fluid under spinal anesthesia with hyperbaric bupivacaine (0.5%) and fentanyl. By assessing electrolyte dynamics in this context, we aim to contribute to the enhancement of perioperative care protocols, thus further optimizing patient outcomes in TURP procedures. The primary outcome of the study was to compare the changes in the level of sodium pre- and postoperatively, whereas, changes in potassium, chloride, and hematocrit were the secondary outcome.

## Materials and methods

This prospective observational study was conducted after obtaining approval from the ethics committee of the Institute of Medical Sciences, Banaras Hindu University (2014-15/EC/1220), and written informed consent from the participants to participate in the study. The study was conducted from July 2015 to December 2017 in the Department of Urology, Institute of Medical Sciences, Banaras Hindu University. Eligible participants included 60 patients aged between 50 and 70 years, with the American Society of Anesthesiologists physical status of 1 and 2. Patients with a history of drug allergy, refusal of operation, spine deformity, bleeding diathesis, pre-existing electrolyte derangements, or severe pre-existing medical conditions were excluded from the study. Prior to the surgical procedure, routine blood investigations were performed, including complete blood count, liver function test, renal function test, electrolytes, random blood sugar, and viral markers.

In the operating room, preoperative baseline parameters, including non-invasive blood pressure (NIBP), heart rate, and oxygen saturation (SpO2), were recorded, and venous access was established on the dorsum of the non-dominant hand and connected to balanced salt solutions. Under strict aseptic conditions, the L3/4 interspace was identified with the patient in the sitting position, and spinal anesthesia was administered using a 25G Quincke’s needle. Confirmation of needle placement was done with the observation of free cerebrospinal fluid flow. Bupivacaine heavy (0.5%) 2 ml and fentanyl 25 micrograms were injected into the subarachnoid space at a rate of 0.2 ml/second, following which the patient was placed in the supine position.

Continuous monitoring of vital parameters was maintained throughout the procedure, with particular attention to the occurrence of hypotension or bradycardia. Hypotension, defined as a decrease in systolic blood pressure (SBP) by more than 30% from baseline or a fall below 90 mmHg, was managed with incremental intravenous doses of mephentermine (5 mg) to maintain SBP above 90 mmHg. Symptomatic bradycardia was treated with intravenous atropine.

Following confirmation of adequate anesthesia and fixation of the desired level, patients were positioned in the lithotomy position for the TURP procedure. The 0.9% normal saline (NS) was used as the irrigating fluid during the surgery to maintain visualization and facilitate tissue removal. After completion of the surgical procedure, patients were transferred to the post-anesthesia care unit (PACU). Serum electrolyte levels were recorded six hours post surgery to assess for any alterations. Dyselectrolytemia was defined as <130 mEq/L and >145 mEq/L for sodium, <3.5 mEq/L or >5.5 mEq/L for potassium, and <96.0 mEq/L and >106.0 mEq/L for chloride. Other parameters recorded were gland size, duration of resection, and volume of irrigation fluids.

The analysis of the data was done with SPSS 15.0 (SPSS Inc., Chicago, IL). Continuous variables were presented as mean and standard deviation while categorical variables were presented in terms of percentage and frequency. After TURP, changes in hematocrit and serum electrolytes like sodium, potassium, and chloride were computed. The paired t-test was employed to compare pre and postoperative hematocrits and serum electrolytes. A p-value of <0.05 was considered significant.

## Results

**Figure 1:**
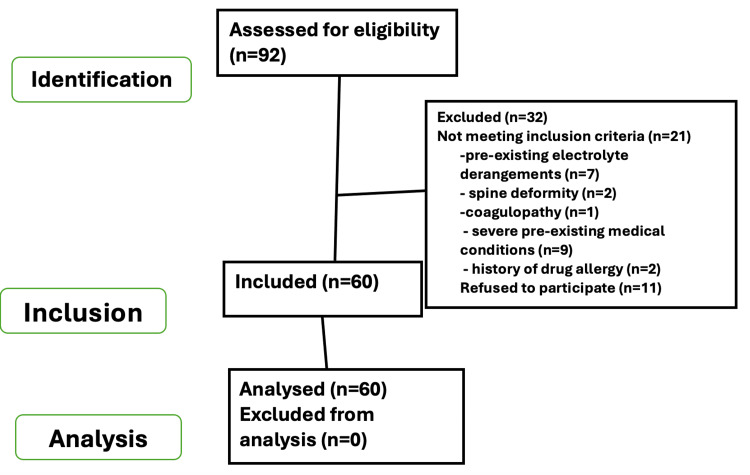
STROBE flow chart STROBE: Strengthening the Reporting of Observational Studies in Epidemiology.

A total of 92 patients were assessed for eligibility, out of which only 60 were included in the study (Figure 1).

The mean age of the patients in our study was 62.92 years (Table 1).

**Table 1:** Baseline parameters The values are mentioned as mean ± standard deviation.

Parameters	Mean ± SD
Age (years)	62.92 ± 6.22
Hematocrit (%)	40.32 ± 6.27
Blood urea (mg/dl)	29.23 ± 8.69
Serum creatinine (mg/dl)	1.08 ± 0.25
Gland size (cc)	61.34 ± 18.67
Resection time (minutes)	35.28 ± 23.22
Volume of irrigation fluid (liters)	14.78 ± 5.60

The preoperative and postoperative mean values of different variables were recorded and analyzed (Table 2).

**Table 2:** Hematocrit and electrolytes The values are mentioned as mean ± standard deviation. * A p-value < 0.05 is statistically significant using a paired t-test.

Variables	Preoperative mean ± SD	Postoperative mean ± SD	p-value
Hematocrit (%)	40.32 ± 6.27	31.07 ± 5.40	<0.001*
Serum sodium (meq/L)	136.77 ± 3.27	128.31 ± 5.91	<0.001*
Serum potassium (meq/L)	4.02 ± 0.26	3.81 ± 0.36	<0.001*
Serum chloride (meq/L)	101.58 ± 2.88	102.25 ± 1.66	0.12*

Preoperative hematocrit was 40.32 and postoperatively it was 31.06. This change in hematocrit was statistically significant, with the p-value being <0.001. The mean preoperative sodium level was 136.77 mEq/L, while postoperatively, it decreased to 128.31 mEq/L (p-value < 0.001). The mean potassium level also changed from 4.02 mEq/L to 3.81 mEq/L postoperatively (p-value < 0.001). Serum chloride level, on the other hand, changed only slightly from 101.58 to 102.25 mEq/L (p > 0.05). Postoperative serum sodium <130 mEq/l, serum potassium <3.5 mEq/l, and serum chloride <96 mEq/l were seen in 43.33%, 25%, and 3.33% of patients, respectively (Table 3).

**Table 3:** Postoperative serum electrolytes mEq/L: milliequivalent per liter; n: numbers.

Serum electrolyte		Frequency (n = 60)	Percentage
Sodium (mEq/L)	>130	34	56.67
	<130	26	43.33
	>145	0	0
Potassium (mEq/L)	>3.5	45	75.0
	<3.5	15	25.0
	>5.5	0	0
Chloride(mEq/L)	>96.0	58	96.67
	<96.0	02	3.33
	>106	0	0

None of the patients had sodium/potassium/chloride greater than 145/5.5/106 mEq/l, respectively.

## Discussion

The changes in serum sodium and potassium were significantly low in the postoperative period with a p-value of <0.001; they did not have any physiological consequences in our study. BPH poses a significant health concern among aging males, affecting nearly half of the male population over 60 years old. TURP stands as the primary endoscopic surgical intervention for managing BPH, albeit with recognized risks, particularly concerning fluid absorption-related complications. These complications often manifest as cardiovascular and neurological symptoms due to electrolyte imbalances resulting from intraoperative fluid absorption.

Bipolar TURP with NS has several benefits, including a lower risk of TURP syndrome, more time for resection, better hemostasis, more surgeon comfort, better surgical exposure with less collateral and penetrative tissue damage, shorter catheter indwelling times, earlier hospital discharge, and higher patient satisfaction [11]. However, a dose-dependent temporary dilution hyperchloraemic acidosis is brought on by the infusion of 0.9% saline solution [12,13].

Our study delved into the perioperative changes in hematological, biochemical, and hemodynamic parameters among patients undergoing TURP, shedding light on the physiological responses and potential complications associated with the procedure. Notably, we observed significant decreases in serum sodium and potassium levels postoperatively, reflecting the dilutional effect of fluid absorption during surgery. Lowering of hematocrit in our study too can be attributed to the dilutional effect of saline irrigation besides intraoperative blood loss. Numerous TURP irrigation fluids have been used, and the effects on changes in serum electrolytes have been thoroughly investigated. In a randomized control trial involving 360 patients undergoing TURP with irrigation fluid consisting of 0.9% saline, 1.5% glucose, or 1.5% glycine, Yousef et al. observed a negligible rise in serum sodium and a decrease in serum potassium in the saline group relative to the other groups [14]. In the glycine group, 17 patients had TURP syndrome, which was not observed in the other two groups. In contrast to other irrigants, the majority of other investigations have discovered a lesser drop in serum sodium levels when NS is used as an irrigating fluid. Additionally, Ho et al. discovered that the mean postoperative serum sodium decreased by 3.2 and 10.7 mmol/L, respectively, for the saline TUR and monopolar TURP groups [15]. Similar drops in sodium levels were observed in many other investigations, including Michielsen et al., Huang et al., and Singhania et al. [16-18].

In our study, there was an insignificant rise in serum chloride levels. There were no differences in serum potassium levels observed by Yousef et al., Hermanns et al., and Singhania et al. from any of the groups, in contrast to our findings [14,18,19].

It is noteworthy that a considerable proportion of patients in our study developed postoperative hyponatremia and hypokalemia, underscoring the need for heightened clinical awareness and proactive measures to prevent electrolyte imbalances in this patient population. While no clinical TURP syndrome was observed in our cohort, the prevalence of electrolyte derangements highlights the potential for adverse outcomes if left unaddressed.

To reduce the incidence of TURP syndrome, various methods have been proposed, including the maintenance of low intravesical pressure, the use of continuous flow resectoscopy, and consideration of alternative surgical techniques such as open surgery or transurethral holmium laser enucleation for patients with larger prostate volumes. Additionally, optimizing surgical parameters such as resection time and the volume of tissue resected may help mitigate the risk of fluid absorption-related complications.

Our study corroborates previous findings regarding the association between tissue resection volume and fluid absorption, emphasizing the importance of careful surgical planning and intraoperative monitoring to minimize complications. Furthermore, attention to factors such as hydrostatic pressure at the prostatic bed and bladder pressure during surgery may further reduce the risk of fluid absorption and its associated complications.

Limitations worth mentioning in our study include that it was an observational study based only on saline irrigation. Other irrigants can be compared in the study. Second, while we looked into the electrolytes, we did not include acid-base alteration that could have also been associated, as seen by Virkar et al. [20]. Third, we did not try to establish a correlation between factors like gland size, duration of resection, and volume of irrigant used with the level of dyselectrolytemia. However, all of these can be covered in subsequent studies.

## Conclusions

TURP is commonly performed in elderly patients with multiple comorbidities, and our findings underscore the importance of comprehensive preoperative assessment and individualized perioperative management to optimize patient outcomes. Although the changes in serum sodium and potassium were statistically significant, they did not have any physiological consequences in our study. While TURP remains an effective treatment option for BPH, clinicians must remain vigilant for potential complications and employ evidence-based strategies to enhance patient safety and minimize morbidity associated with the procedure.
